# Progression of Plasmodium berghei through Anopheles stephensi Is Density-Dependent

**DOI:** 10.1371/journal.ppat.0030195

**Published:** 2007-12-28

**Authors:** Robert E Sinden, Emma J Dawes, Yasmene Alavi, Joanna Waldock, Olivia Finney, Jacqui Mendoza, Geoff A Butcher, Laura Andrews, Adrian V Hill, Sarah C Gilbert, María-Gloria Basáñez

**Affiliations:** 1 Division of Cell and Molecular Biology, Faculty of Life Sciences, Imperial College London, London, United Kingdom; 2 Department of Infectious Disease Epidemiology, Faculty of Medicine, Imperial College London, London, United Kingdom; 3 Wellcome Trust Centre for Human Genetics, University of Oxford, United Kingdom; University of Minnesota, United States of America

## Abstract

It is well documented that the density of *Plasmodium* in its vertebrate host modulates the physiological response induced; this in turn regulates parasite survival and transmission. It is less clear that parasite density in the mosquito regulates survival and transmission of this important pathogen. Numerous studies have described conversion rates of *Plasmodium* from one life stage to the next within the mosquito, yet few have considered that these rates might vary with parasite density. Here we establish infections with defined numbers of the rodent malaria parasite Plasmodium berghei to examine how parasite density at each stage of development (gametocytes; ookinetes; oocysts and sporozoites) influences development to the ensuing stage in Anopheles stephensi, and thus the delivery of infectious sporozoites to the vertebrate host. We show that every developmental transition exhibits strong density dependence, with numbers of the ensuing stages saturating at high density. We further show that when fed ookinetes at very low densities, oocyst development is facilitated by increasing ookinete number (i.e., the efficiency of ookinete–oocyst transformation follows a sigmoid relationship). We discuss how observations on this model system generate important hypotheses for the understanding of malaria biology, and how these might guide the rational analysis of interventions against the transmission of the malaria parasites of humans by their diverse vector species.

## Author Summary

Malaria, one of the world's most devastating parasitic diseases, is caused by protozoan parasites of the genus *Plasmodium* and is transmitted between mammalian hosts by *Anopheles* mosquitoes*.* Within the mosquito, the parasite undergoes four sequential developmental transformations as it passes from the bloodmeal through the mosquito's midgut epithelium to the salivary glands, from where the parasite is inoculated when the mosquito bites the vertebrate host. This study demonstrates, in a laboratory model, that parasite input density at every developmental stage in the mosquito regulates output to the ensuing form. Statistical models were fitted to experimental data to identify and describe the most appropriate functional relationships. In all cases, the relationships between two consecutive parasite stages can saturate at high parasite densities, suggesting that at high parasite densities parasite numbers may have to be reduced substantially to effect an appreciable decrease in parasite transmission. These results may help establish a rational basis for new studies on species of medical importance and further our understanding of how interventions designed to reduce parasite survival within the mosquito might be expected to impact upon transmission.

## Introduction

The availability of the genomes of man, the mosquito and the malarial parasite has enabled penetrating new studies on the molecular organization of *Plasmodium* in its two hosts. Yet, we do not fully comprehend how parasite population densities may affect transmissibility. Without this knowledge our understanding of the impact of host responses, or of external intervention, upon the transmission of the parasite through endemic populations will remain incomplete.

**Figure 1:**
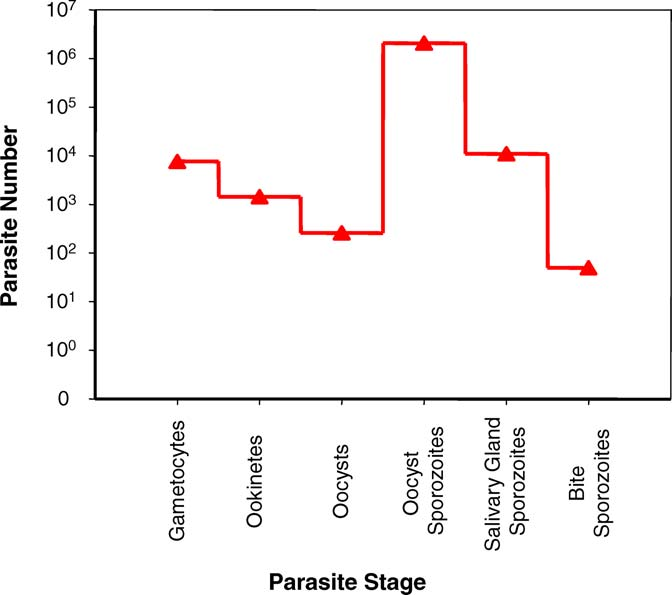
Changes in Parasite Abundance during Development within the Mosquito Reported changes in numbers of P. berghei as it develops in *An. stephensi*, starting from an intake of 10^4^ macrogametocytes. Note the use of a log-scale on the *y*-axis. Figure adapted from Sinden [87], based on data from Alavi et al. [55].

Within the mosquito, *Plasmodium* transforms from macrogametocyte to ookinete, oocyst, and finally to sporozoite. The many studies reporting marked fluctuations in parasite numbers during this development have been elegantly summarized by Vaughan [1]. In susceptible mosquitoes it is the oocyst that frequently represents the nadir of parasite numbers in the life cycle (Figure 1). Within the oocyst, parasite numbers reportedly increase by two or three orders of magnitude [2,3] before the daughter sporozoites make their inefficient passage to the salivary glands. Few are inoculated into the vertebrate host when the female mosquito takes a subsequent bloodmeal [4–8]. In a number of well characterized parasite–mosquito combinations, ookinetes completely fail to cross the midgut epithelium or sporozoites fail to invade the salivary glands [9,10]. An important question that has eluded enquiry, however, is whether the ookinete-oocyst bottleneck and the other developmental transitions through the mosquito are density-dependent. (In this context, a transition is density-dependent when the rate at which such process occurs is determined by the parasite density of the previous stage.) Contributory factors for this omission may include the facts that most previous studies have compared parasite numbers in just two life stages, looked at a single infection intensity, or investigated densities within narrow ranges [11–13].

In this paper we have attempted to address this question by measuring the relationships between wide-ranging densities of successive life stages (macrogametocytes, ookinetes, oocysts and salivary gland sporozoites) achievable in the laboratory model Plasmodium berghei–Anopheles stephensi, and by statistically fitting functional forms to these relationships. Determining the form of these relationships may increase our understanding both of the processes regulating the transmission of this parasite by the mosquito, and (by extrapolation) of the potential impact of intervention measures. Although the biological bases for the parameters thus estimated can be inferred, we make no attempt to verify them experimentally. We recognize that they may include, among others: interspecific competition between the parasite and its vertebrate and invertebrate hosts (the former being largely, though not exclusively, confined to the mosquito bloodmeal, and the latter mediated, for instance, by immune attack from the mosquito); intraspecific competition between parasites; and the possible “altruistic” apoptotic death of the parasite [14]. The incorporation of the functional forms that we report in this paper into a mathematical model describing the transition of *Plasmodium* within the mosquito, and its linkage to malaria models that take into account parasite density in the human host [15], will be presented elsewhere.

## Results

The following results describe the dynamics of P. berghei within *An. stephensi* observed in one laboratory, with experiments conducted by many different researchers on numerous occasions. We recognize that some of the parasite densities achievable in this parasite–insect combination lie outside those normally recorded in most infections of human parasites in their natural vectors; nonetheless, these “extreme values” permit a more objective fitting of functional forms that span the full range of values anticipated in the latter species.

### Estimating the Type and Severity of Density Dependence

The fitting procedures investigated the frequency distribution among mosquitoes of the outcome variable (parasite numbers of the ensuing stage); fitted an appropriate distribution (usually overdispersed); explored the relationship between the degree of overdispersion and mean parasite density; and fitted models to both the means (to allow comparisons to be made with the literature) and to the individual parasite counts (to allow maximum use of the data available) for the relationships between two consecutive parasite stages.

Density dependence can be positive (facilitation) or negative (limitation), characterized by the per capita parasite yield increasing or decreasing, respectively, with parasite density. Initial facilitation may be followed by subsequent limitation, producing a sigmoid relationship. Absence of density dependence, where the rate of success is constant with parasite density is characterized by proportionality. To encompass all these possible behaviors, we fitted the following generalized formula,

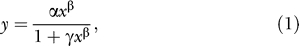
where *x* represents the input and *y* the output parasite density. This function can describe a linear (*α* > 0; *β* = 1; *γ* = 0); a saturating (*α* > 0; *β* = 1; *γ* > 0); or a sigmoid (*α* > 0; *β* > 1; *γ* > 0) relationship, with each function being nested into the following one. The attributes of each of these functional forms are described in Table 1.

**Table 1:**
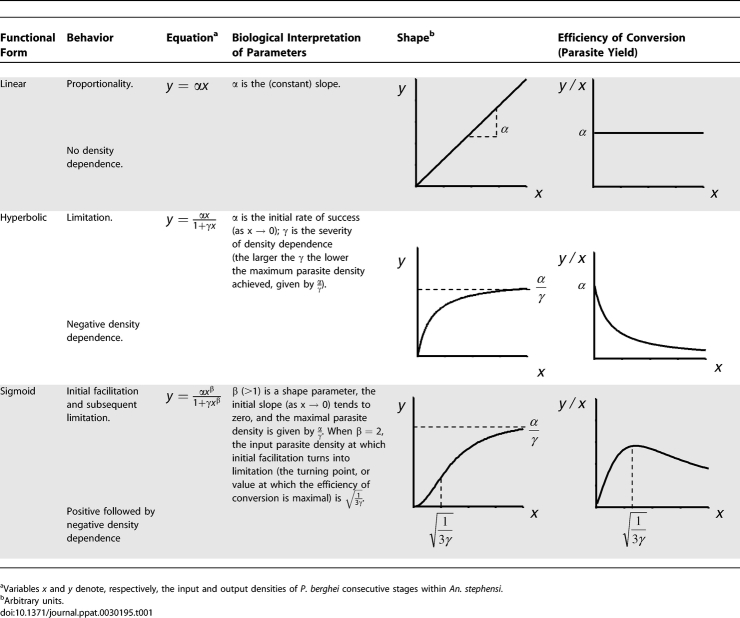
Description of Functional Forms Nested within Equation 1

### Macrogametocyte to Ookinete Transition

**Figure 2:**
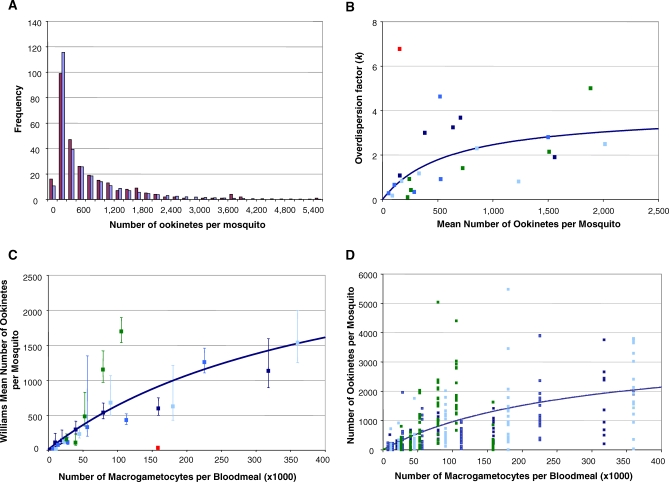
Analysis of Parasite Densities in the Transition from Macrogametocytes to Ookinetes (A) Frequency distribution of the number of ookinetes per mosquito. Observed frequency (red bars), expected frequency according to negative binomial distribution (blue bars) with *k* = 0.45 (an inverse measure of the degree of overdispersion; i.e., the lower the value of *k*, the greater the departure from the Poisson, random distribution), estimated by maximum likelihood. (B) Maximum likelihood estimates of parameter *k* (for each macrogametocyte density) against arithmetic mean ookinete density. The degree of overdispersion decreases (*k* increases) with mean ookinete density with a saturating relationship when the outlying red point (from OF's experiments) is excluded. (C) WM number of ookinetes per mosquito against number of macrogametocytes per bloodmeal. Ookinete density increases nonlinearly with macrogametocyte density when the outlying red point (from JM's experiment) is excluded. Error bars denote standard errors of WMs. (D) Number of ookinetes per individual mosquito against number of macrogametocytes per bloodmeal. The fitted curve corresponds to a saturating function (with underling nonlinear relationship between overdispersion parameter and mean ookinete density). All data pertain to P. berghei in *An. stephensi*. Markers in (B–D) refer to three experiments by OF (blue) and one by JM (green).

Data for this transition originated from two researchers on four separate occasions. The behavior of this transition was independent of the worker conducting the infections (results not shown). Analysing the datasets jointly revealed that the overall distribution of the number of ookinetes per mosquito was strongly overdispersed, confirmed by a variance over mean ratio (VMR) of 1108 (where a VMR of 1 suggests a Poisson, random distribution). A negative binomial distribution (NBD) was fitted to the overall frequency of mosquitoes harboring a given number of ookinetes; this revealed an arithmetic mean infection of 643 ookinetes per mosquito and an overdispersion parameter estimate of 0.45 (95% confidence interval (CI): 0.02–3.59) (Figure 2A). The chi-square goodness of fit test indicated good agreement between the observed and expected distributions (χ^2^ = 13.2, degrees of freedom (df) = 12, *p* = 0.36). When a separate NBD was fitted for each macrogametocyte density and its parameters estimated, the most overdispersed distributions were found among the lowest ookinete densities, and the degree of overdispersion decreased with increasing density (Figure 2B) as found in other parasite–vector systems [16].

Due to the overdispersion found in the data, the relationship between the number of macrogametocytes offered in the bloodmeal, and the resulting mean ookinete density, was investigated using the geometric mean of Williams (WM) [17] as a measure of central tendency for ookinete density. Excluding one outlying datapoint (where exceptional parasite death occurred) the best-fitting expression proved to be a saturating, hyperbolic function (with *α* = 0.008 (0.006–0.009) and *γ* = 2.36 × 10^−6^ (1.09 × 10^−6^–4.27 × 10^−6^)), indicating that the efficiency of this transition declines monotonically with increasing macrogametocyte density (Figure 2C). Initially ∼125 macrogametocytes are required to produce one ookinete and ookinete numbers saturate at a value (given by *α/γ*) of ∼3,500 per mosquito.

A hyperbolic relationship between the number of ingested macrogametocytes and the resulting numbers of ookinetes produced, was again indicated when plotting the ookinete counts for individual mosquitoes (with *α* = 0.013 (0.012–0.014) and *γ* = 3.57 × 10^−6^ (2.56 × 10^−6^–4.67 × 10^−6^); Figure 2D). This again suggests that the probability of a macrogametocyte becoming an ookinete declines with increasing input. This analysis predicts that initially ∼77 macrogametocytes make one ookinete, and this relationship again saturates at a density of ∼3,500 ookinetes per mosquito.

### Ookinete to Oocyst Transition

Data for this transition came from three researchers and nine experiments. We recognize that the following analysis may be influenced by the atypical relationship that might exist between the pre-formed ookinetes in the bloodmeal and the midgut of the insect. As in the previous transition, oocyst numbers were overdispersed (VMR = 63). An NBD was fitted to the combined datasets, and to the oocyst frequency distributions for each ookinete input density. However in this case, the NBD was not the most appropriate to describe the frequency of mosquitoes with a given oocyst density (χ^2^ = 158.8, df = 29, *p* < 0.001) (unlike [18]); this may be because our ookinete feed technique (unlike the gametocyte feeds used in [18]), rarely fails to infect mosquitoes. As in the previous transition, the degree of overdispersion was maximal at low oocyst mean densities, and decreased with increasing density.

**Figure 3:**
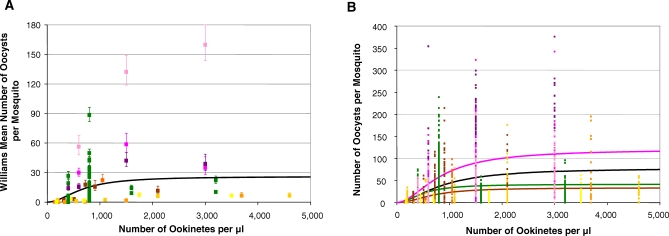
Analysis of Parasite Densities in the Transition from Ookinetes to Oocysts (A) WM number of oocysts per mosquito against number of ookinetes per μl of blood. Mean oocyst density is related to ookinete density by a sigmoid function. Error bars denote standard errors of the WMs. (B) Number of oocysts per individual mosquito against number of ookinetes per μl of blood. The black line is fitted to the combined data; the brown line to data from [88]; the green line to data from JM; and the pink line to data from experiments by JW. In all cases the fitted curve corresponds to a sigmoid function (with an underlying nonlinear, power relationship between overdispersion and mean oocyst density). Assuming a bloodmeal volume of 2.13 μl, ookinete density can be expressed per mosquito by multiplying by 2.13. All data pertain to P. berghei in *An. stephensi*. Markers refer to one experiment by JM (green), three by JW (pink/violet), and five by [88] (orange/yellow/brown).

The fitted relationship between input ookinete density and the resulting WM oocyst number (Figure 3A) indicated a sigmoid relationship, suggesting the operation of initial facilitation and subsequent limitation (with *α* = 6.87 × 10^−5^ (5.28 × 10^−5^–8.85 × 10^−5^); *β* = 1.98 (1.89–2.11) and *γ* = 2.64 × 10^−6^ (1.88 × 10^−6^–3.81 × 10^−6^)). Initially, 120 ookinetes produce one oocyst; at the point of maximum yield ∼60 ookinetes produce one oocyst; and as the number of ookinetes increases further, the transformation becomes constrained above a mean density of ∼26 oocysts per mosquito.

As with the relationship identified for the means, the best fitting model for the number of oocysts per individual mosquito as a function of ookinete density (Figure 3B), was sigmoid (with parameters *α* = 5.84 × 10^−4^ (5.55 × 10^−4^, 6.12 × 10^−4^); *β* = 1.74 (1.73, 1.76); γ = 7.44 × 10^−6^ (6.89 × 10^−6^, 8.11 × 10^−6^)). Initially ∼72 ookinetes are required to produce one oocyst; at the point of maximum yield this requirement decreases to ∼35 ookinetes per oocyst; and the relationship saturates at a maximum of ∼80 oocysts per mosquito as the number of ookinetes increases. When testing inter-experimenter variation it was clear that even in the same laboratory, oocyst production from ookinete membrane-feeds varied markedly between different researchers. Nonetheless each experimenter's data invariably indicated that, despite the differing overall efficiency of oocyst production, every relationship was sigmoid (Figure 3B, Table 2).

**Table 2:**
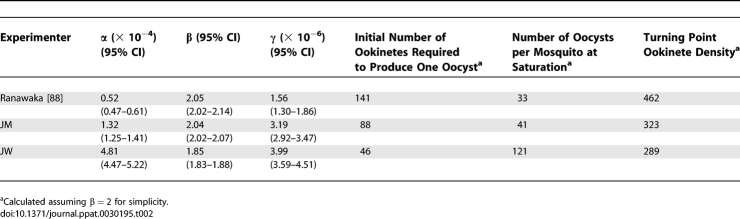
Parameter Estimates for Sigmoid Models Fitted to the Ookinete to Oocyst Transition

Recognizing that the transformation of the ookinete into an oocyst is dependent not only on the ookinete locating and invading the midgut wall, but also on the ability of the intracellular ookinete to survive attack by the mosquito's innate immune mechanisms, we have, in replicate studies, examined directly the ability of green fluorescent protein (GFP)-expressing ookinetes to invade the gut cells. Following the ingestion of 3,600 GFP-tagged ookinetes, a mean of 516 (or 14%) were detected 24 hours later in the midgut epithelium. This number of ookinetes would be expected to produce 80 oocysts (Figure 3B) and thus at this infection intensity just 16% (80/516) of the ookinetes in the midgut epithelium are predicted to be detected as oocysts 9 days later.

### Oocyst to Sporozoites in the Salivary Glands

Data for this transition came from three independent experiments by one researcher. The number of sporozoites per mosquito showed strong overdispersion (VMR = 1,393). The distributions resulting from each oocyst density were separately examined for an NBD, and again, overdispersion decreased with increasing sporozoite density. However, as for the frequency distribution of oocysts, the NBD did not fit the distribution of the number of salivary gland sporozoites per mosquito (χ^2^ = 41.3, df = 19, *p* < 0.002) as well as it did for the ookinete distribution.

**Figure 4:**
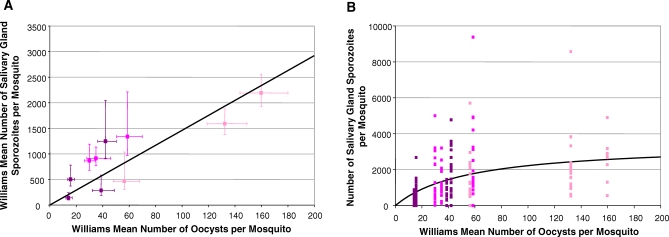
Analysis of Parasite Densities in the Transition from Oocysts to Sporozoites in the Salivary Glands (A) WM number of sporozoites in the salivary glands against WM number of oocysts per mosquito. Mean sporozoite density increases linearly with oocyst density. Error bars denote standard errors of the WMs. (B) Number of salivary gland sporozoites per individual mosquito against the WM number of oocysts per mosquito. The fitted curve corresponds to a saturating function (with underling linear relationship between overdispersion and mean ookinete density). All data pertain to P. berghei in *An. stephensi*. Markers refer to three experiments by JW.

The relationship between the output WM number of salivary gland sporozoites per mosquito and the input WM oocyst density per mosquito (Figure 4A) was most parsimoniously fitted by a linear model (with *α* = 14.61 (11.58–17.63)) based on the likelihood ratio statistic (LRS) analyses, although this model was only marginally better than the hyperbolic fit. (The latter was suggested as the better model according to the Akaike Information Criterion (AIC) and this discrepancy may indicate insufficient power to distinguish between the two models.) This relationship predicts that in this study on average, each oocyst produces between just 12 and 18 sporozoites that successfully invade the salivary glands. This relationship became significantly nonlinear when analyzing the number of salivary gland sporozoites per individual mosquito as a function of the mean oocyst density (Figure 4B). In this case, the best fitting model was hyperbolic (with *α* = 62.21 (53.98–72.20) and *γ* = 0.018 (0.013–0.024)), indicating that initially 54–72 salivary gland sporozoites are produced per oocyst, and thereafter the efficiency of conversion declines with rising oocyst density, reaching a plateau of ∼3,500 salivary gland sporozoites per mosquito irrespective of rising oocyst input.

## Discussion

### The Role of Experimental Models

We recognize that one model system cannot accurately reflect the diverse biology of the hundreds of natural malaria–vector combinations found worldwide; nonetheless we also recognize that studies on the biology of Plasmodium spp., and their interactions with Anopheles spp., have been advanced considerably by the analysis of malaria parasites of rodents. Exploiting the rare opportunity to study, in a controlled environment, cloned populations of P. berghei in an inbred line of *An. stephensi* at widely differing parasite densities has permitted us to raise questions in the model that are orders of magnitude more difficult to study in the parasites of man. Under these specific conditions, we show that parasite's developmental transitions within the mosquito are density-dependent. These conclusions are consistent with limited (mainly laboratory based) studies on the malaria parasites of humans (see below). They help generate key hypotheses that now require to be tested on other parasite–vector combinations. We recognize that future laboratory studies on , for example, *P. falciparum–An. gambiae*, would have to conduct experiments with lab adapted strains of both parasite and vector; not only are these experiments more costly (in addition to requiring extensive safety management), but also the lab strains themselves lack the diversity of natural populations. We recognise therefore that the key studies will be those on the parasites and vectors in their numerous and different endemic areas. If validated in the human malarias, the hypotheses generated in this study may have important implications for the design of anti-malarial intervention programs.

To our knowledge, this is the first quantitative investigation of the impact of parasite density upon all transitions between *Plasmodium* stages within the mosquito. Our data have permitted analyses not only of measures of central tendency in groups of mosquitoes (the most frequent type of (aggregate) analysis reported in the literature), but also of parasite counts in individual mosquitoes, and their distribution.

### Parasite Distribution

Gametocyte numbers in the bloodmeal are variously described as being normally distributed [1,19] or overdispersed [20,21]; ookinete numbers in the ingested bloodmeal as being normally distributed [1] or overdispersed [19,22]; and oocysts and salivary gland sporozoites as being markedly overdispersed [6,18,19,22,23]. Recognising that the NBD can originate, for instance, when each host /vector is infected according to a Poisson process whose mean is gamma-distributed (i.e., there is marked heterogeneity in mosquito susceptibility [18]), we were interested to find that even using highly “inbred” organisms, ookinete, oocyst, and gland sporozoite numbers per mosquito exhibit strong overdispersion (see also [24,25]). As previously observed [16,18], the severity of overdispersion was itself density-dependent, decreasing with mean parasite density both when the relationship between overdispersion and parasite load was analyzed separately (having fitted distributions to each parasite density previously), and when the degree of overdispersion was allowed to vary with parasite density whilst jointly fitting models to individual parasite counts.

### Oocyst Development within the Mosquito

In designing these experiments we were cognizant of early studies which recognized that infectivity of individual gametocyte carriers differs widely over the course of an infection [26–28]. Whereas P. berghei ookinete production *in vitro* faithfully reflected gametocyte density in the blood, oocyst formation in the mosquito host was severely compromised after day 5 of the blood infection [27]. Whereas some prior studies have concluded that gametocyte-oocyst intensities are linearly related [11,12], others noted a nonlinear relationship in P. falciparum within, but not between experiments [29]. Ponnudurai et al. [29] and Pichon et al. [30] reported the operation of density-dependent suppression of oocyst development in P. falciparum; we have similarly found that the gametocyte-oocyst transformation is density-dependent. Notwithstanding the operation of nonlinearities, the efficiency of this conversion in our experimental *P. berghei–An. stephensi* system tends to be lower than that estimated in the natural combinations *P. gallinaceum–Aedes aegypti* [11] and *P. falciparum–An. gambiae* [12] (results not shown).

The numerous blood-borne variables responsible for modulating gametocyte-oocyst development *in vivo* transcend species, and many suppress the early conversion of gametocytes to ookinetes [31–40]. Mosquito factors regulating infection are less well understood but include physiological, immunologic and biotic variables [41–44], the expression of which varies with both mosquito and parasite species [45,46], as well as with genotypes [31,47]. Interestingly, it has been suggested that *Plasmodium* may have immunosuppressive effects upon the vector [48]. Consistent with the above, recent analyses on P. vivax concluded that it is vertebrate factors that impact largely upon fertilization, whereas mosquito factors determine ookinete losses [49].

Unlike these previous studies which investigated the relationship between gametocytes and oocysts (and found or not some evidence of nonlinearity), our work has aimed at teasing out where exactly nonlinearities may be occurring, and therefore we have examined the transition from gametocytes to ookinetes separately from that of ookinetes to oocysts. In P. falciparum the efficiency of macrogametocyte to ookinete conversion *in vivo* reportedly varies widely (from 0.025% to 42%) [50]. In P. berghei, we find that the efficiency of conversion for the transition of macrogametocytes to ookinetes is maximal at the lowest parasite densities (i.e., the hyperbolic models were found to best fit this relationship). At the lowest macrogametocyte densities investigated (i.e., ∼7,000 per bloodmeal) the efficiency of ookinete production *in vivo* is ∼1.3%; thereafter the efficiency falls progressively with increasing density (0.6% for 360,000 macrogametocyes/bloodmeal). This fall suggests competition for limited resources, or density-dependent stimulation of a parasite-killing response in the mosquito [44,51,52].

Interestingly, when examining ookinete to oocyst development we found a sigmoid relationship, indicating that at low ookinete densities, and with this experimental design, the transition was positively density-dependent (i.e., the per capita probability of successful oocyst establishment was very low at the lowest ookinete densities but increased initially with increasing ookinete density in the bloodmeal). Similarly, a sigmoid zygote-oocyst relationship was reported by Rosenberg et al. when P. gallinaceum female zygotes produced *in vitro* were membrane-fed to *Ae. aegypti* [53]. The maximal parasite yield (100%) was achieved for zygote densities of ∼4/mosquito, but at ∼40,000/mosquito the per zygote efficiency had decreased to 0.3%. A possible biological explanation for this initial facilitation may lie in the difficulty that individual ookinetes may have to disrupt the mosquito's peritrophic matrix or midgut epithelial cells, whereas at higher ookinete densities, those which succeed in penetrating these structures may make it easier for other ookinetes to do so, facilitating their passage and ultimately their establishment under the basal lamina of the midgut epithelium. These results are entirely consistent with an earlier study by Munderloh and Kurtti [54], who observed that low numbers of P. berghei ookinetes do not reliably produce oocyst infections. Assuming a bloodmeal volume of 2.13 μl [19], our data suggest that to ensure infection with at least one oocyst, approximately 40–140 ookinetes/mosquito are required (Table 2), whereas they estimated that ∼1,100 (purified) ookinetes would be necessary. We attribute these differences in number to recent improvements in the parasite culture. Studies on *P. falciparum in vivo* [19] similarly indicate an apparent “threshold” ookinete density of 30/mosquito in *An. gambiae*. Sigmoid relationships have a “turning” parasite density at which the maximum probability of transformation is achieved and beyond which negative density dependence operates. The turning-point ookinete density (assuming *β* = 2 for simplicity) is 355 for the model fitted to mean P. berghei oocyst density (Figure 3A), and 212 for the model fitted to all individual data combined (black curve in Figure 3B; see Table 2 for researcher-specific turning points). Other examples of initial facilitation followed by subsequent limitation within a vector have been found among filarial parasites [16]. Earlier studies [50,55–57] remarked on the high cost of ookinete-oocyst transformation, and suggest it to be a critical block. Published work suggests that at this stage of development, mosquitoes can be significantly (*P. falciparum / An. albimanus* [19]; *P. yoelii / An. albimanus* [22,58]) or totally refractory (*P. berghei / Ae. Aegypti* [55]). However, in compatible parasite–vector combinations, e.g., *P. falciparum / An. freeborni* [19] and *P. yoelii / An. stephensi* [22], transformation can be efficient and parasite losses at the late ookinete stage rare. It will be interesting to explore whether the specificity and/or magnitude of the mosquito's immune responses are sensitive to ookinete density. It is known that immune responses are qualitatively different in different parasite–vector combinations [59], and their cost to vector fitness and survival is not insignificant [48,60–62].

### Oocyst Maturation and Sporozoite Delivery

The transition from oocyst to salivary gland sporozoite is usually inefficient and can be totally inhibited in some *Plasmodium*/mosquito combinations [3]; whether sporozoites released from the oocyst are removed from the hemocele by hemocytes, or lysed by immune peptides is still unknown [63]. Salivary gland burden is markedly increased if oocyst-infected mosquitoes take a second, uninfected bloodmeal, typically 4 days after infection [64]. This may overcome inter-oocyst competition for nutrients, but it has been suggested that it synchronizes sporozoite maturation [29,65]. Whilst some authors suggest that mosquito survivorship is not adversely impacted by oocyst density [10,66], others found that mosquito mortality increased with oocyst burden [67,68]. Our experimental design was not influenced by these variables; the experiments were all subjected to the same (single) blood-feed regimen, and all examined mosquitoes were alive immediately prior to dissection.

The mathematical relationship between abdominal oocysts and salivary gland sporozoites is complicated by the variable and unknown sporozoite production within oocysts [64,69,70]. Previous studies have reported that the number of sporozoites produced per oocyst is not density-dependent [22], and studies on P. vivax [13,66] reported linear relationships between oocyst and salivary gland sporozoite prevalences. Others have described only a weak (Pearson) correlation between oocyst, and sporozoite number in the glands in both P. falciparum, and P. vivax [10]. Geometric mean oocyst loads of 2.6 (1–197) and 2.2 (1–26) in, respectively, naturally infected *An. gambiae* and *An. funestus* have been correlated with mean loads of 962 and 812 salivary gland sporozoites. Assuming a production of ∼10,000 sporozoites per oocyst [2], this would translate in only 4% of sporozoites invading the salivary glands [23]. Our data suggest that mean oocyst numbers are linearly related to mean salivary gland sporozoite load (Figure 4A, *r* = 0.9), but that a saturating (hyperbolic) relationship best describes the sporozoite numbers per individual mosquito (Figure 4B). At most naturally occurring oocyst burdens (1–5 per mosquito), our results suggest that the number of sporozoites in the glands is most likely to be directly proportional to oocyst load, as found with P. falciparum in *An. gambiae*, however the latter combination exhibits a much higher efficiency (i.e., ∼400–700 P. falciparum gland sporozoites/oocyst [23,50] versus 54–72 P. berghei gland sporozoites/oocyst). However at the high oocyst numbers (>50) achievable in *P. berghei / An. stephensi,* it is evident that salivary gland numbers are rate-limited (Figure 4B).

**Figure 5:**
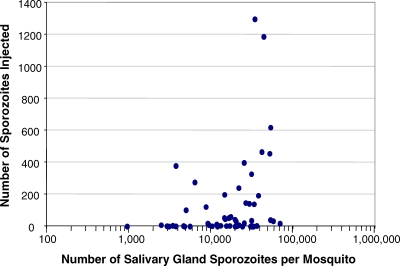
Relationship between the Number of Salivary Gland Sporozoites and Sporozoites Transferred in the Bite Data from Medica and Sinnis [6] (P. yoelii in *An. stephensi*) illustrating the relationship between the number of sporozoites counted in dissected salivary glands and the number of sporozoites observed in the saliva ejected by a single bite. Note the use of a logarithmic scale on the *x*-axis.

Previous authors have discussed, for P. berghei, P. yoelii, and P. falciparum [2,5–9,71–74], the importance of sporozoites being located in the salivary gland ducts at the time of feeding in relation to the probability of a sporozoite being inoculated into the skin of the host. Figure 5 illustrates the enormous variation reported in sporozoite inocula in the bites of *An. stephensi* with wide-ranging salivary gland burdens of sporozoites of the rodent malarias. Whilst it is widely conjectured that sporozoites of different Plasmodium spp. differ dramatically in their infectivity to their vertebrate hosts, published and unpublished data kindly made available to us (Table 3) suggest that for all species studied, inocula of just 10 sporozoites can be infectious. It is therefore relevant to investigate the question “At what salivary gland sporozoite density, will the number of sporozoites in the bite fall below 10?” Whilst the data suggest gland burdens as high as 30,000 can result in inocula below this “threshold”, it is clear that gland infections of just a few hundred sporozoites (that could be derived from 1–2 oocysts in the *P. falciparum / An. gambiae* combination) have clear infection potential. It is thus obvious that the prevalence and not the intensity of oocysts or salivary gland sporozoite infections will be the key practical parameter when considering the potential infectivity of individual mosquitoes to the vertebrate host.

**Table 3:**
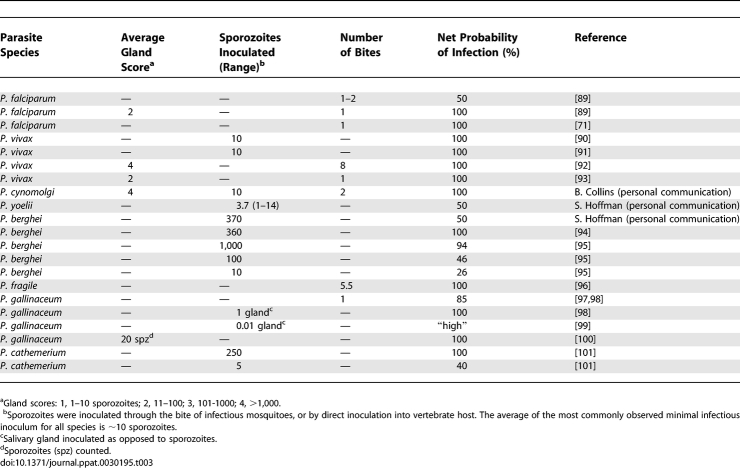
Number of Sporozoites Required to Establish a Patent Parasitaemia in Vertebrate Hosts

### Implications for the Modeling of Transmission-Blocking Strategies

Our overarching goal is to develop mathematical models of the population biology of malaria within the mosquito that ultimately relate the prevalence and intensity of gametocytaemia in the vertebrate host with the entomological inoculation rate and the force of infection, and to link these frameworks with novel models of malaria in the human host [15]. Thus far, these studies have found little relationship between the infectiousness of human populations to vectors and the resulting transmission intensity from vectors back to humans.

**Figure 6:**
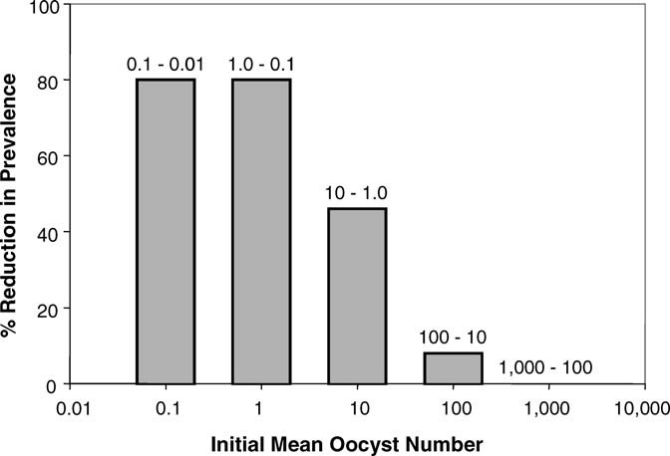
Reduction in Oocyst Prevalence Induced by a 90% Reduction in Intensity at Different Initial Densities A 90% blockade in intensity at varying oocyst numbers by a theoretical intervention results in the greatest reductions in prevalence of infected mosquitoes when mean oocyst numbers are low. Data from Medley et al. [18].

The very poor correlation between salivary gland burden and sporozoite inoculum at the next bite, suggests that reductions in oocyst number may not correlate well with the potential impact of intervention upon transmission. In contrast, it is abundantly clear that any effective transmission-blocking strategy will have to reduce both oocyst intensity and prevalence (their inter-relationship being nonlinear [18]). The biological consequences of reductions in prevalence cannot be contested —uninfected mosquitoes cannot transmit. The varying reduction in prevalence that would be induced, by a 90% reduction in oocyst intensity, at different initial oocyst densities is illustrated in Figure 6. If studies into transmission-blocking strategies were to discuss their efficacy in terms that unequivocally reduce the number of infectious bites (and therefore the force of infection), as argued by those applying anti-vector policies, they might facilitate the wider understanding and acceptance of the obvious impact of such interventions in endemic communities. We will report elsewhere how our data will permit us to forward hypotheses as to how interventions targeted at different mosquito stages, e.g., gametes, ookinetes, oocysts or sporozoites, might be expected to reduce the prevalence of infectious mosquitoes in a theoretical population.

## Materials and Methods

### The parasite.

To eliminate the impact of host and parasite genetic variability, parasite clones, and one inbred mosquito line were used. All but one experiment reported here used clone 234 of the rodent malarial parasite P. berghei strain ANKA. The parasite was maintained by serial passage, but no more than eight sequential mechanical blood passages took place before passage through mosquitoes [27,33]. This regimen maintains gametocyte infectivity to the mosquito, a critical property for this study. Gametocyte density and male: female ratios were determined in Giemsa stained smears, and infections were always done on days 3–5 when a low but rising gametocytaemia prevailed [27,33]. Male:female ratios invariably fell within the normal range for low-passage P. berghei infections, i.e., 1.64 ± 0.93SD (Dearsly, unpublished). All details of direct, or membrane feeds and parasite enumeration are as described previously [75]. Those experiments in which there was significant insect mortality were excluded, on the understanding that this phenomenon may itself be related to infection intensity [76]. In an effort to facilitate parasite identification, one set of experiments used the GFP-expressing transgenic clone (PbGFPCON) [77] derived from the HP line of P. berghei. Experiments counting ookinetes in the bloodmeal used the indirect fluorescent antibody test (IFAT) to reveal parasites expressing P28/Pbs21 [78]. In all calculations it has been assumed that the bloodmeal volume in A. stephensi is 2.13 μl [19].

### The hosts.

All gametocytes were raised in Theiler's Original (TO) mice and transmitted to A. stephensi strain Sd 500, maintained at 19 °C and 80% RH, and fed on 5% fructose/0.05% para-amino-benzoic acid as described previously [75].

### Experimental design.

The impact of numerous extraneous factors (e.g., host serum; mosquito midgut milieu) upon gametocyte infectivity complicates the design and reproducibility of experiments in P. berghei [34,27]. Pools of gametocytes were therefore washed free of variable serum factors and re-suspended in a single pool of serum known to support P. berghei infectivity. Gametocytes, re-suspended at known parasite densities, were fed to replicate aliquots of A. stephensi from the same rearing brood. Fifteen hours later, GFP-expressing, or P28-positive retorts and ookinetes were counted by IFAT in dissected bloodmeals. Ten days after blood feeding, oocysts were counted by phase contrast microscopy of freshly dissected midguts. Sporozoites were similarly counted on days 21–25 by hemocytometry of dissected salivary glands. Unless otherwise stated, all experiments were repeated in triplicate and all mosquitoes examined were alive immediately prior to dissection. We have not attempted to study oocyst infections below a mean of 10 as the proportion of uninfected mosquitoes rises rapidly making the necessary group sizes unmanageably large [18]. To study the impact of ookinete density upon oocyst formation we exploited our ability to culture P. berghei ookinetes in vitro. Following culture ookinetes were resuspended at known densities in fresh heparinised mouse blood and fed by membrane feeder [75]. Under these conditions it is anticipated that some ookinetes will invade the midgut epithelium earlier than when mosquitoes are infected by gametocytes.

Even with the advent of GFP-tagged parasite lines it is not possible to count midgut oocysts in vivo without compromising the subsequent development of the sporozoites in the mosquito, due to direct UV irradiation, or stress/damage to the insect. Thus, batches in excess of 200 mosquitoes were each infected with different ookinete numbers such that different oocyst burdens could be determined by dissecting groups of at least 50 mosquitoes [79]. The remainder of each mosquito batch (>50) was incubated a further 8–15 days before counting the salivary gland sporozoites. With this design, both input and output parasite densities are random variables.

### Sporozoite counting by polymerase chain reaction (PCR).

Efforts to enumerate sporozoites in the saliva secreted into a bloodmeal, using PCR, were not successful, and have been superseded by the elegant studies of others [5,6,72]. However, for the sake of completeness, we give an account of our procedures in Text S1.

### Statistical methods.

*Frequency distributions.* Frequency distribution histograms were prepared for each of the output life stages of interest, i.e., the number of ookinetes, the number of oocysts, and the number of salivary gland sporozoites per mosquito. The VMR was calculated for each distribution, which indicated the presence of overdispersion (the ratio was significantly greater than 1 as ascertained by chi-square tests [80]). An NBD (whose parameters are the arithmetic mean, and the exponent, *k*, an inverse measure of the degree of overdispersion) was fitted to the data using maximum likelihood (ML). The goodness of fit of the negative binomial distribution to the observed frequency distribution was assessed using chi-square tests [80]. For each life stage, values for *k* were thus estimated for the combined data, and for each input parasite density separately, and plotted against the corresponding arithmetic mean number of parasites to assess whether the overdispersion parameter was independent or related to the means of the distributions. The functional form of the relationship between *k* and the mean was determined by fitting various models (detailed in Text S2) using ML, which were compared using the LRS [81] for nested models and the AIC [82] for non-nested models. The LRS and AIC results can be found in Table S1, and the resulting parameter values in Table S2.

*Models fitted to mean parasite densities.* Functional relationships were fitted to mean parasite densities as a function of the (pre-defined) previous life stage density, using the WM [17] as a measure of central tendency for the outcome variable (given the overdispersed nature of the data as assessed above). Asymmetric CIs were estimated for the WMs [83]. When the explanatory variable was also the mean of a random variable (rather than a pre-determined parasite density fed to mosquitoes), CIs were also calculated for the variable plotted on the horizontal axis. Heterogeneity in the data between experiments and between workers was examined to determine whether a single model should be fitted to the entire dataset, to individual experiments, or to experimenter subsets. The form of the relationship between two subsequent life stages, using the mean values, was determined by fitting Equation 1 by ML using quasi-Newton algorithms. We maximized a sample size-weighted log-likelihood for a normally distributed variable after applying a suitable (square root) transformation of the means. The weighting procedure allowed us to take into account the number of mosquitoes contributing to each mean value in addition to the total number of mean values [84]. In this case, *y* was the WM of the outcome variable and *x* was the parasite density of the previous (input) life stage. Parameters *α*, *β*, and *γ* determine the shape of the functional form as described in Table 1, and the resulting models were compared using the LRS [81] as the models were nested (linear versus hyperbolic and versus sigmoid; hyperbolic versus sigmoid). (Details of the LRS results are provided in Table S3.) Asymptotic 95% CIs [85] were estimated for each parameter in the final model.

*Models fitted to individual parasite densities.* In order to make better use of the data available, models were also fitted to individual parasite densities rather than the mean values, using parasite densities from individual mosquitoes against the pre-defined (or mean) parasite densities of the previous life stage. The form of the relationship between two subsequent life stages using the individual values was determined by fitting the functional form given in Equation 1, where *x*, *α*, *β*, and *γ*, were as described above, and *y* was the parasite count of the subsequent stage as observed in individual mosquitoes. Parameters were estimated, using quasi-Newton algorithms, by maximising a negative binomial log-likelihood that allowed the overdispersion parameter to be a function of the mean [86]. Again, models were compared using LRS, and asymptotic 95% CIs [85] were estimated for the parameters in the final model, including overdispersion parameters. Details of model comparison can be found in Table S4. In all cases *p* < 0.05 was considered to indicate a significant departure from the null hypothesis in question. Limitations of the statistical analyses are discussed in Text S3.

## Supporting Information

Table S1 Model Comparisons for Relationship between Overdispersion Parameter and Mean Parasite Density (54 KB DOC)

Table S2 Parameters of the Most Parsimonious Models for Relationships between Overdispersion and Mean Parasite Density (28 KB DOC)

Table S3 Model Comparisons for Relationship between Output Mean Parasite Density and Parasite Density of the Preceding (Input) Life Stage (39 KB DOC)

Table S4 Model Comparisons for Relationship between Output Individual Parasite Density and the Parasite Density of the Preceding (Input) Life Stage (38 KB DOC)

Text S1 Sporozoite Counting by PCR (24 KB DOC)

Text S2 Parasite Overdispersion (25 KB DOC)

Text S3 Limitations of the Statistical Analysis (25 KB DOC)

## Abbreviations

AIC: Akaike Information Criterion
CI: confidence interval
df: degrees of freedom
IFAT: indirect fluorescent antibody test
GFP: green fluorescent protein
LRS: likelihood ratio statistic
ML: maximum likelihood
NBD: negative binomial distribution
PCR: polymerase chain reaction
WM: Williams' mean
VMR: variance over mean ratio
