# Supplementary material for: Progression of Plasmodium berghei through Anopheles stephensi Is Density-Dependent
Source: PLoS Pathog. 2007 Dec 28;3(12):e195. doi: 10.1371/journal.ppat.0030195 (PMC2156095; doi:10.1371/journal.ppat.0030195)
Supplement: Table S1 — (54 KB DOC) [file ppat.0030195.st001.doc]

**Table S1. Model Comparisons for Relationship between Overdispersion Parameter and Mean Parasite Density**

| **Comparison** | | **Sigmoid** | | | **Hyperbolic** | | | **Power** | | | **Linear** | | |  |
| --- | --- | --- | --- | --- | --- | --- | --- | --- | --- | --- | --- | --- | --- | --- |
| **Model** | ***L*** | **df** | **LRS** | ***p*** | **df** | **LRS** | ***p*** | **df** | **LRS** | ***p*** | **df** | **LRS** | ***p*** | **AIC** |
| **Parameter *k* as a function of ookinete density** | | | | | | | | | | | | | | |
| Sigmoid  **Hyperbolic**  Power  Linear  Constant | -34.659  -35.089  -35.518  -37.295  -41.452 | 1  1  2  3 | 0.860  1.716  5.272  13.584 | 0.354*  0.190*  0.072*  0.004* | 1  2 | 4.412  12.724 | 0.036*  0.002* | 1  2 | 3.555  11.868 | 0.059*  0.003* | 1 | 8.313 | 0.004* | 77.319  76.179  77.035  78.590  86.903 |
| **Parameter *k* as a function of oocyst density** | | | | | | | | | | | | | | |
| Sigmoid  Hyperbolic  **Power**  Linear  Constant | -19.97  -23.87  -19.97  -23.87  -43.49 | 1  1  2  3 | 7.797  0  7.797  47.024 | 0.005*  0.991*  0.020*  <0.001* | 1  2 | <0.001  39.227 | 0.978*  <0.001* | 1  2 | 7.797  47.024 | 0.005*  <0.001* | 1 | 39.226 | <0.001* | 49.949  55.746  47.950  51.747  92.973 |
| **Parameter *k* as a function of sporozoite density** | | | | | | | | | | | | | | |
| Sigmoid  Hyperbolic  Power  **Linear**  Constant | -12.208  -12.208  -12.213  -12.216  -13.692 | 1  1  2  3 | 0.001  0.010  0.016  2.969 | 0.977*  0.920*  0.992*  0.397* | 1  2 | <0.001  2.9680 | 0.903*  0.227* | 1  2 | 0.006  2.959 | 0.940*  0.228* | 1 | 2.953 | 0.086* | 34.416  32.417  32.426  28.432  33.385 |

*L,* Log-likelihood; df, Degrees of Freedom; LRS, Likelihood Ratio Statistic; AIC, Akaike Information Criterion.

* indicates a significant *p* value, i.e. that the model being tested (indicated in the column heading) is significantly better than the null model (indicated in the corresponding row heading).

Models in bold font indicate the most parsimonious yet adequate model according to LRS (for nested models) and AIC (for non-nested models).
