# Supplementary material for: Progression of Plasmodium berghei through Anopheles stephensi Is Density-Dependent
Source: PLoS Pathog. 2007 Dec 28;3(12):e195. doi: 10.1371/journal.ppat.0030195 (PMC2156095; doi:10.1371/journal.ppat.0030195)
Supplement: Table S2 — (28 KB DOC) [file ppat.0030195.st002.doc]

**Table S2. Parameters of the Most Parsimonious Models for Relationships between Overdispersion and Mean Parasite Density**

| **Relationship** | **Most Parsimonious Model** | **Parameter Values** (95% CI) |
| --- | --- | --- |
| ***k* as a function of ookinete density** | Hyperbolic ^‡^ | *k_1_* = 0.0065 (0.0051–0.0079)  *k_3_* = 0.0016 (0.0012–0.0024) |
| ***k* as a function of oocyst density** | Power | *k_0_* = 0.5363 (0.4347–0.6387)  *k_1_* = 3.07x10^-4^ (2.53x10^-4^–3.61x10^-4^)  *k_2_* = 1.8082 (1.7698–1.8408) |
| ***k* as a function of sporozoite density** | Linear ^‡^ | *k_1_* = 8.41x10^-4^ (5.41x10^-4^–11.41x10^-4^) |

CI, Confidence Interval.

* See equation 2 for the corresponding functional form of the model.

^‡^  *k_0_* (intercept) parameter omitted as not significantly different from zero (not shown).
