# Supplementary material for: Progression of Plasmodium berghei through Anopheles stephensi Is Density-Dependent
Source: PLoS Pathog. 2007 Dec 28;3(12):e195. doi: 10.1371/journal.ppat.0030195 (PMC2156095; doi:10.1371/journal.ppat.0030195)
Supplement: Table S3 — (39 KB DOC) [file ppat.0030195.st003.doc]

**Table S3.**  Model Comparisons for Relationship between Output Mean Parasite Density and Parasite Density of the Preceding (Input) Life-stage

| **Comparison** | | **Sigmoid** | | | **Hyperbolic** | | |  |
| --- | --- | --- | --- | --- | --- | --- | --- | --- |
| **Model** | ***L*** | **df** | **LRS** | ***p*** | **df** | **LRS** | ***p*** | **AIC** ^‡^ |
| **WM ookinete density as a function of macrogametocyte density** | | | | | | | | |
| Sigmoid  **Hyperbolic**  Linear | -71.939  -72.317  -74.314 | 1  2 | 0.756  4.750 | 0.385*  0.093* | 1 | 3.994 | 0.046* | 156.628  150.634  152.628 |
| **WM oocyst density as a function of ookinete density** | | | | | | | | |
| **Sigmoid**  Hyperbolic  Linear | -166.727  -168.646  -174.226 | 1  2 | 3.837  14.997 | 0.050*  0.001* | 1 | 11.160 | 0.001* | 341.455  343.292  352.451 |
| **WM salivary gland sporozoite density as a function of WM oocyst density** | | | | | | | | |
| Sigmoid  Hyperbolic  **Linear** | -72.199  -72.288  -74.065 | 1  2 | 0.177  3.732 | 0.674*  0.155* | 1 | 3.555 | 0.059* | 152.398  150.575  152.130 |

Footnotes as in Table S1.

^‡^  As all models are nested, model comparisons are based on the LRS results. AIC values are given here for the sake of completeness.

The parameter values and 95% CIs for the models in bold font are given in the main text.
