# Supplementary material for: Progression of Plasmodium berghei through Anopheles stephensi Is Density-Dependent
Source: PLoS Pathog. 2007 Dec 28;3(12):e195. doi: 10.1371/journal.ppat.0030195 (PMC2156095; doi:10.1371/journal.ppat.0030195)
Supplement: Table S4 — (38 KB DOC) [file ppat.0030195.st004.doc]

**Table S4.**  Model Comparisons for Relationship between Output Individual Parasite Density and the Parasite Density of the Preceding (Input) Life-stage

| **Comparison** | | **Sigmoid** | | | **Hyperbolic** | | |  |
| --- | --- | --- | --- | --- | --- | --- | --- | --- |
| **Model** | ***L*** | **df** | **LRS** | ***p*** | **df** | **LRS** | ***p*** | **AIC** ^‡^ |
| **Ookinete density as a function of macrogametocyte density** | | | | | | | | |
| Sigmoid  **Hyperbolic**  Linear | -2779.75  -2781.55  -2786.00 | 1  2 | 3.594  12.508 | 0.058*  0.002* | 1 | 8.914 | 0.003* | 5567.500  5569.107  5576.007 |
| **Oocyst density as a function of ookinete density** | | | | | | | | |
| **Sigmoid**  Hyperbolic  Linear | -11852.89  -11930.87  -11956.86 | 1  2 | 155.963  207.933 | <0.001*  <0.001* | 1 | 207.933 | <0.001* | 23713.778  23867.741  23917.710 |
| **Salivary gland sporozoite density as a function of WM oocyst density** | | | | | | | | |
| Sigmoid  **Hyperbolic**  Linear | -1532.71  -1533.80  -1539.17 | 1  2 | 2.184  12.927 | 0.139*  0.002* | 1 | 10.742 | 0.001* | 3074.417  3073.601  3082.344 |

Footnotes as in Table S1.

^‡^  As all models are nested, model comparisons are based on the LRS results. AIC values are given here for the sake of completeness.

The parameter values and 95% CIs for the models in bold font with overdispersion as a function of mean parasite density are given in the main text.
