# Supplementary material for: Progression of Plasmodium berghei through Anopheles stephensi Is Density-Dependent
Source: PLoS Pathog. 2007 Dec 28;3(12):e195. doi: 10.1371/journal.ppat.0030195 (PMC2156095; doi:10.1371/journal.ppat.0030195)
Supplement: Text S1 — (24 KB DOC) [file ppat.0030195.sd001.doc]

**Text S1: Sporozoite counting by PCR.**

We include here a brief critique of our unsuccessful methodology for the information of others. Quantitative real-time PCR used previously published primers targeting the multi-copy 18S ribosomal RNA genes of all *Plasmodium* species [Andrews L, Andersen RF, Webster D, Dunachie S, Walther RM, et al. (2005) Quantitative real-time polymerase chain reaction for malaria diagnosis and its use in malaria vaccine clinical trials. Am J Trop Med Hyg 73: 191-198]. Purified *P. berghei* sporozoites were freeze-thawed three times before DNA extraction (DNA BloodMini kit, QIAGEN) and 18S rRNA PCR. The PCR product was cloned into a standard plasmid vector (pCR-BluntII-TOPO, Invitrogen) and quantified by spectrophotometry. A PCR standard curve was produced using plasmid dilutions of 10 to 106 equivalent genome copies. Known numbers of *P. berghei* sporozoites from dissected *An. stephensi* salivary glands were diluted in an erythrocyte solution to provide samples of known concentration. DNA extractions were carried out as described above for 18S rRNA PCR, blinded to source. The genome copy number standard curve was used to estimate sporozoite concentrations. No correlation was found between estimated sporozoite number by PCR and original sporozoite counts.
