# Supplementary material for: Progression of Plasmodium berghei through Anopheles stephensi Is Density-Dependent
Source: PLoS Pathog. 2007 Dec 28;3(12):e195. doi: 10.1371/journal.ppat.0030195 (PMC2156095; doi:10.1371/journal.ppat.0030195)
Supplement: Text S2 — (25 KB DOC) [file ppat.0030195.sd002.doc]

**Text S2: Parasite overdispersion.** The functional form of the relationship between the negative binomial overdispersion parameter *k* and the arithmetic mean of each distribution of output stages, $\bar{y}$, was explored by fitting the following expression by maximum likelihood (ML) assuming normality of errors,

, (2)

which allows constant *k* $(k=k_{0};k_{1}=0)$ to be compared with linear $(k_{2} =1,k_{3}=0)$; power $(k_{3}=0)$; hyperbolic $(k_{2} =1,k_{3}>0)$; and sigmoid $(k_{1}>0, k_{2} >1, k_{3}>0)$ functions of the mean, using the likelihood ratio test statistic (LRS) [81] when the models were nested, or the Akaike Information Criterion (AIC) when they were not [82] (e.g. when comparing power and hyperbolic models). Model comparison results for each of the transitions are presented in Table S1. Asymptotic 95% confidence intervals [85] were estimated for each parameter in the most parsimonious (yet adequate) model (Table S2).
