# Supplementary material for: Progression of Plasmodium berghei through Anopheles stephensi Is Density-Dependent
Source: PLoS Pathog. 2007 Dec 28;3(12):e195. doi: 10.1371/journal.ppat.0030195 (PMC2156095; doi:10.1371/journal.ppat.0030195)
Supplement: Text S3 — (25 KB DOC) [file ppat.0030195.sd003.doc]

**Text S3: Limitations of the statistical analysis.**

Two important limitations of our statistical study merit discussion. The first stems from the fact that mosquito batches were infected through membrane feeders by provision of blood with the estimated, given parasite density. It is possible that the infection status of mosquitoes belonging to the same replicate was more similar than that of mosquitoes of other replicates, and therefore individual parasite counts for mosquitoes infected through the same membrane feeder may not be truly independent. This could result in our standard errors being somewhat underestimated and the severity of density dependence overestimated. The second limitation applies in particular but not solely to the transition from oocysts to salivary gland sporozoites, since due to the necessity of having to study two separate samples of the same mosquito batch both variables are subject to measurement error. The (unaccounted) presence of measurement error in the explanatory variable my accentuate nonlinearity in regression analyses [Carroll RJ, Ruppert D, Stefanski LA (1995) Measurement error in nonlinear models. Chapman and Hall]. Further work will therefore be necessary to account for the possible intra-correlation structure in the data and to evaluate the influence of measurement error on our parameter estimates. Although our strongly nonlinear results regarding the ookinete to oocyst transition will probably be robust to departures from the independence assumption and unlikely to be greatly influenced by measurement error, the true degree of nonlinearity in the macrogametocyte to ookinete, and oocyst to sporozoite transitions is likely to be weaker than that reported here. The results of these investigations will be presented elsewhere.
